# Efficacy and safety of osimertinib for leptomeningeal metastases from EGFR-mutant non-small cell lung cancer: a pooled analysis

**DOI:** 10.1186/s40001-023-01219-y

**Published:** 2023-08-04

**Authors:** Lei Wen, Junjie Zhen, Changguo Shan, Mingyao Lai, Weiping Hong, Hui Wang, Mingting Ye, Yanying Yang, Shaoqun Li, Zhaoming Zhou, Jiangfen Zhou, Qingjun Hu, Juan Li, Xuwei Tian, Longhua Chen, Linbo Cai, Zhanhong Xie, Cheng Zhou

**Affiliations:** 1 Department of Oncology, Guangdong Sanjiu Brain Hospital, Guangzhou, China; 2 Department of Radiation Oncology, The First People’s Hospital of Kashi Prefecture, Kashi, China; 3 Department of Radiation Oncology, Nanfang Hospital, Southern Medical University, Guangzhou, 510515 People’s Republic of China; 4 State Key Laboratory of Respiratory Disease, National Clinical Research Center for Respiratory Disease, Guangzhou Institute for Respiratory Health, The First Affiliated Hospital of Guangzhou Medical University, Guangzhou, 510120 People’s Republic of China

**Keywords:** Osimertinib, EGFR, NSCLC, Leptomeningeal metastases (LM), Efficacy, Meta-analysis

## Abstract

**Background:**

The aim of this study was to evaluate the efficacy and safety of osimertinib for the treatment of leptomeningeal metastases (LM) from epidermal growth factor receptor (EGFR)-mutant non-small cell lung cancer (NSCLC).

**Methods:**

We conducted a systematic review and meta-analysis to aggregate the clinical outcomes of patients with LM from EGFR-mutant NSCLC treated with osimertinib. A comprehensive literature search for published and unpublished studies was implemented in April 2021 of PubMed, EMBASE, the Cochrane Library, and several international conference databases, in accordance with the PRISMA guidelines. Meta-analysis of proportions was conducted to calculate the pooled rate of overall response rate (ORR), disease control rate (DCR), one-year overall survival (OS), and adverse events (AEs).

**Results:**

A total of eleven studies (five prospective and six retrospective) including 353 patients were included. The majority of patients (346/353, 98.0%) received osimertinib as ≥ 2nd-line treatment for LM, either at a dosage of 80 mg (161/353, 45.6%) or 160 mg (191/353, 54.1%). The pooled rates of ORR and DCR were 42% (95% CI 24% to 59%) and 93% (95% CI 88% to 97%), respectively. The pooled one-year OS rate was 59% (95% CI 53% to 65%) in 233 patients from five studies. The highest incidence of AEs of all grades was rash (53%), followed by diarrhea (45%), paronychia (35%), decreased appetite (35%), and dry skin (27%), based on data from four studies.

**Conclusions:**

Our study highlighted and confirmed the meaningful efficacy and a manageable safety profile of osimertinib for the treatment of LM from EGFR-mutant advanced NSCLC.

**Supplementary Information:**

The online version contains supplementary material available at 10.1186/s40001-023-01219-y.

## Introduction

Leptomeningeal metastases (LM), the spread of tumor cells into the leptomeninges and cerebrospinal fluid, represents one of the most deleterious complications of advanced non-small cell lung cancer (NSCLC) [1]. LM is associated with dismal prognosis due to poor response to cytotoxic agents and radiation treatment [2]. Of note, with emergence of modern treatment options such as targeted therapy and immunotherapy, considerably prolonged survival was achieved in patients with NSCLC. Owning to the rapid advances in imaging techniques and particularly tyrosine kinase inhibitors (TKIs) for patients harboring epidermal growth factor receptor (EGFR) mutations, recent years saw increasing incidence of LM from NSCLC [3].

Osimertinib is a third-generation EGFR TKI that inhibits both EGFR sensitizing and T790M mutations in NSCLC. Compared with first- and second-generation EGFR TKIs, osimertinib is more effectively cross the blood–brain barrier (BBB) and therefore with a superior cerebrospinal fluid (CSF) plasma concentration [4]. The phase III FLAURA study demonstrated higher efficacy of osimertinib compared to gefitinib or erlotinib for advanced NSCLC patients harboring EGFR-sensitizing mutations, including patients with central nervous system (CNS) metastases [5, 6]. However, the FLAURA study included only five patients with LM but not the patients with unstable neurological conditions. The AURA LM study reported the activity of osimertinib (80 mg) for the treatment of LM from NSCLC in a retrospective pooled analysis of four prospective studies within the AURA program (AURA extension, AURA2, AURA17, and AURA3) [7]. The included 22 LM patients, and the LM objective response rate (ORR), median LM progression-free survival (PFS) and overall survival (OS) were 55%, 11.1 months, and 18.8 months, respectively. The phase I BLOOM study evaluated the efficacy and safety of double dosage of osimertinib (160 mg qd) in forty-one NSCLC patients with LM [8]. The LM ORR assessed by the Response Assessment in Neuro-Oncology LM (RANO-LM) criteria was 62%. The median investigator-assessed PFS was 8.6 months, and the median OS was 11.0 months. Preliminary conclusions from these studies outlined a promising role of osimertinib for the treatment of LM from EGFR-mutant (EGFRm) NSCLC.

Nevertheless, many published articles or meeting abstracts on osimertinib for the treatment of LM from NSCLC enrolled a relatively small number of patients (< 50 patients in most studies), which weakens the persuasiveness of the conclusions from a single study. Furthermore, those studies vary in study design (prospective *versus* retrospective), dosages (80 mg *versus* 160 mg), race (Asian *versus* Caucasian), or LM response assessment criteria (RANO-LM *versus* Response Evaluation Criteria in Solid Tumors (RECIST)). Herein, an aggregate analysis of the currently available reports is highly demanded to clarify the role of osimertinib in management of LM from EGFRm NSCLC. In our study, we synthesized the outcomes from different studies with a particular focus on LM ORR, LM DCR, PFS, OS, and AEs, providing more insights for the optimal clinical use of osimertinib. This systematic review and meta-analysis was carried out according to the Preferred Reporting Items for Systematic Reviews and Meta-analysis (PRISMA) guidelines [9].

## Materials and methods

### Literature search strategy

We searched for available articles, either published literatures or in conference abstracts with respect to evaluations of the safety and efficacy of osimertinib for EGFR-mutated NSCLC with leptomeningeal metastases. First, a comprehensive literature search on PubMed, EMBASE, and the Cochrane Library was conducted in April 2021 using the following retrieval strategy: (“osimertinib” OR “mereletinib” OR “AZD9291” OR “tagrisso”) AND (“leptomeningeal metastases” OR “leptomeningeal metastasis” OR “carcinomatous meningitis” OR “CNS” OR “central nervous system”). Secondly, meeting abstracts from the three international oncology conferences (American Society of Clinical Oncology (ASCO), European Society of Medical Oncology (ESMO), and World Conference on Lung Cancer (WCLC)) from 2015 to 2020 were screened. Finally, reference lists of retrieved or relevant studies were also inspected to identify additional articles.

### Selection criteria

Two authors independently screened titles and abstracts, and disagreement was resolved by a third author. After preliminary screening, the full texts of potentially eligible studies were reviewed to confirm final inclusion using the following selection criteria: (1) prospective clinical trials, retrospective cohort series, or prospective cohort series were available; (2) human studies were performed to evaluate the efficacy and safety of osimertinib for the treatment of patients with LM (diagnosed cytologically and/or radiologically) from EGFRm NSCLC. (3) The studies were published in English, and the most complete and recent report of the trial was used when the same team reported data obtained from the same patients. (4) Regarding the number of patients, due to the relatively low incidence of LM, an enrollment of at least 5 patients from prospectively designed trials or at least 10 patients from retrospective cohorts were required. Case reports, review articles, animal experiments, and duplicate publications were excluded.

### Data extraction

Data were extracted and filled in a standardized, predesigned Microsoft Excel form by 2 investigators independently. All relevant data from texts, tables, and figures of each included study were extracted, including first author, publication year, region, number of patients, study design, age, sex, dosage, treatment line of osimertinib, EGFR T790M status, response assessment criteria, follow-up, LM ORR, LM DCR, PFS, OS, and adverse events (AEs), etc. If the response of LM was assessed both by neuroradiologic blinded central in dependent review (BICR) and by investigator, the BICR reported data were preferably recorded. If the prognosis was plotted as a Kaplan–Meier curve in some articles, the software GetData Graph Digitizer 2.24 (http://getdata-graph-digitizer.com/) was applied to digitize and extract the data of one-year OS from the survival curve. Any discrepancies were resolved by discussion or by consulting a third investigator when necessary.

### Quality assessment

The modified Newcastle–Ottawa Scale (modified NOS) was used to evaluate the quality of all included articles due to the single arm and non-controlled design [10, 11]. Compared to the NOS, modified NOS eliminates three questions: two relating to selection and comparison of nonexposed patients and one assessing the presence of outcome at study start, and adds one question addressing presence of pharmaceutical industry funding. In brief, the modified NOS includes 6 appraisal items: representativeness of exposed cohort, ascertainment of exposure, assessment of outcome, median follow-up more than 6 months, follow-up completeness, and pharmaceutical industry funding [11].

### Statistical analysis

The primary outcome of our study was LM ORR, which was evaluated by the response assessment criteria for LM in the study. Secondary outcomes included LM DCR, one-year OS rate, median LM PFS, median OS, and AEs. The integrated analysis of ORR, DCR, and one-year OS rate was carried out using the generic inverse variance random-effect method, and the effect size was represented by the 95% confidence interval (CI). Heterogeneity between studies was assessed using the Cochran Q statistic and I^2^. Heterogeneity was considered high, medium or low if ≥ 75%, 50–75%, or < 50%, respectively [12]. A fixed or random effect model was used depending on heterogeneity analysis. AEs of all grades or of grade ≥ III were aggregated separately. Furthermore, funnel plots were used to assess the publication bias of the enrolled studies. All statistical analyses were performed with R version 4.0.4 (R Foundation, Vienna, Austria) using the meta package. A two-sided p-value of < 0.05 was considered as statistically significant.

## Results

### Study selection

**Fig. 1:**
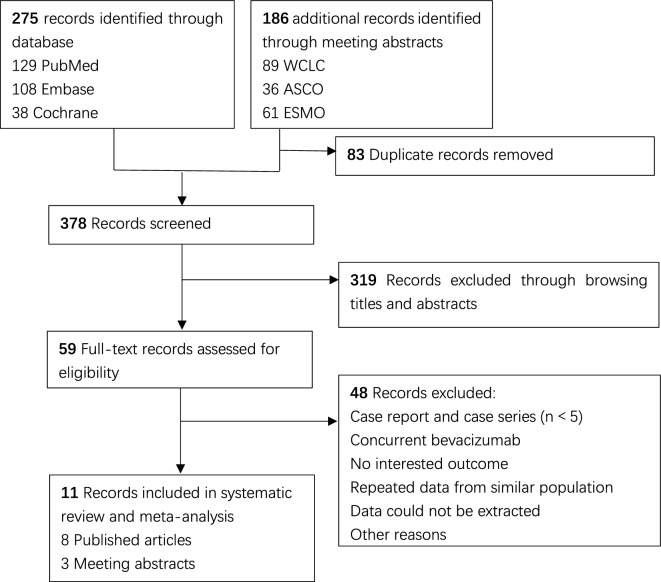
Preferred Reporting Items for Systematic Reviews and Meta-analyses (PRISMA) Flow Diagram

The study flowchart is provided (Fig. 1). In total, 461 studies were identified after performing database searches (275 for PubMed, Embase, and Cochrane Library, 186 for WCLC, ASCO, and ESMO), and 378 studies remained after the removal of duplicates. A total of 319 records were excluded after screening of the titles and abstracts. Finally, another 11 studies (including 8 published articles [6–8, 13–17] and 3 meeting abstracts [18–20]) were included after reading of the full texts.

### Study characteristics

The 11 included records involving 353 NSCLC patients with LM who received osimertinib. Those records were published between 2017 and 2020 and consisting of 1 phase I study (BLOOM) [8], 3 phase II studies [14, 18, 19], 1 prospective pilot study [15], 1 preplanned exploratory analysis of a phase III study (FLAURA) [6], 1 retrospective pooled analysis of 4 prospective studies [7], and 4 retrospective cohort studies [13, 16, 17, 20]. A majority of patients (346/353, 98.0%) received osimertinib as ≥ 2nd-line treatment for LM, either at a dosage of 80 mg (161/353, 45.6%) or 160 mg (191/353, 54.1%). LM response assessment criteria were available in 7 studies, including 3 that used RANO-LM criteria, 2 that used RECIST 1.1 criteria, and 2 that used radiological response (nonspecific). Follow-up data were available for 5 studies, with a follow-up period between 8.3 and 11.7 months. The patient characteristics of the included studies were summarized in Table 1.

**Table 1:** Characteristics of 11 included studies

Study (year)	Region	No. of patients (female %)	Study design	Age	Therapy line	Dosage of Osimertinib (mg)	T790M status	LM response assessment criteria	Follow-up (mo.)
Yang 2019	Asian	41 (71%)	Phase I	59 (44–75)	≥ 2	160 mg	Positive:22; negative:13; unknown:6	RANO-LM (BICR)	9.9
Lee 2020	Asian	110 (62%)	Retrospective	58 (39–75)	≥ 2	80 mg (n = 67); 160 mg (n = 43)	Positive:60; negative:37; unknown:13	NA	NA
Ahn 2020	Global	22 (59%)	Retrospective pooled analysis of 4 prospective studies	58 (36–80)	≥ 2	80 mg	100% positive	RANO-LM (BICR)	11.7
Park 2020	Asian	40 (60%)	Phase II	59 (38–77)	≥ 2	160 mg	100% positive	RECIST 1.1	9.6
Nanjo 2017	Asian	13 (62%)	Prospective pilot study	67 (54–79)	≥ 5	80 mg	100% positive	CNS radiographic change	NA
Zheng 2020	Asian	45 (60%)	Retrospective	54 (22–82)	1(16%); ≥ 2(84%)	80 mg	Positive:9; negative:36	RANO-LM	NA
Saboundji 2018	Europe	20 (70%)	Retrospective	61.2 (11.2)	≥ 2	80 mg (17); 160 mg (2); 40 mg (1)	65% positive	NA	11.1
Reungwetwattana 2018	Global	5 (NA)	Preplanned, exploratory analysis of FLAURA	NA	1	80 mg	0%	RECIST 1.1	NA
Akazawa 2019	Asian	6 (50%)	Phase II	61.5 (50–75)	≥ 2	80 mg	100%	Radiological response	NA
Ahn 2019	Asian	40 (NA)	Phase II	NA	≥ 2	80 mg: 16; 160 mg: 24	100%	NA	8.3
Andrew 2020	American	11	Retrospective	NA	≥ 2	160 mg	NA	NA	NA

BICR: blinded central in dependent review; CNS: central nervous system; LM: leptomeningeal metastases; mo.: months; NA: not available; RANO: Response Assessment in Neuro-Oncology; RECIST: Response Evaluation Criteria in Solid Tumors

### Pooled LM ORR and DCR

**Fig. 2:**
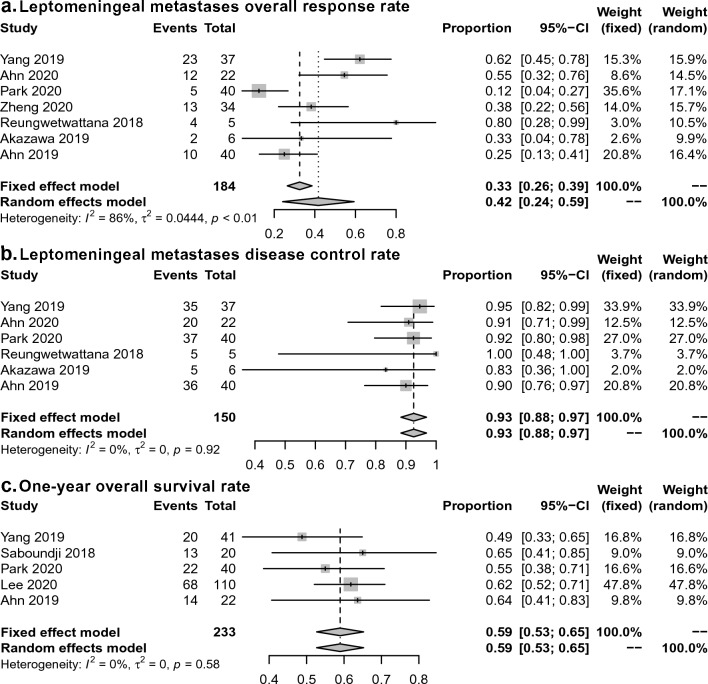
Meta-analysis of ORR, DCR, and one-year OS of osimertinib for the treatment of LM from EGFR-mutant NSCLC. **a** ORR; **b** DCR; **c** one-year OS

LM ORR was available in 7 studies [6–8, 14, 16, 18, 19], and LM DCR was available in 6 studies [6–8, 14, 18, 19]. The pooled LM ORR was 42% (95% CI 24% to 59%; I^2^ = 86% according to the random-effects model; Fig. 2a), and the pooled LM DCR was 93% (95% CI 88% to 97%; I^2^ = 0% according to the fixed-effects model; Fig. 2b).

The pooled LM ORR and DCR were similar when excluding studies published in abstract form, at 47% (95% CI 23% to 72%; I^2^ = 90%) for ORR and 95% (95% CI 91% to 100%; I^2^ = 0%) for DCR. When excluding 2 studies with a small number of patients (n < 10), the pooled LM ORR and DCR were found to be 38% (95% CI 19% to 57%; I^2^ = 88%) and 92% (95% CI 88% to 97%; I^2^ = 0%), respectively.

In the subgroup analysis, the pooled LM ORR was 33% (95% CI 5% to 60%; I2 = 93%) in three prospective phase I/II studies, which was lower than the 50% (95% CI 32% to 67%; I^2^ = 46%) of the four retrospective cohort studies. With respect to the dosage of osimertinib, the pooled LM ORR was 47% (95% CI 36% to 59%; I^2^ = 46%) for patients treated with 80 mg osimertinib and 37% (95% CI 0% to 86%; I^2^ = 96%) for patients treated with 160 mg osimertinib. The funnel plots were roughly symmetric for ORR and DCR (Additional file 1: Fig. S1). This indicated no publication bias and objectively reporting by the included studies.

### 1 year OS rate, median PFS, and OS

**Fig. 3:**
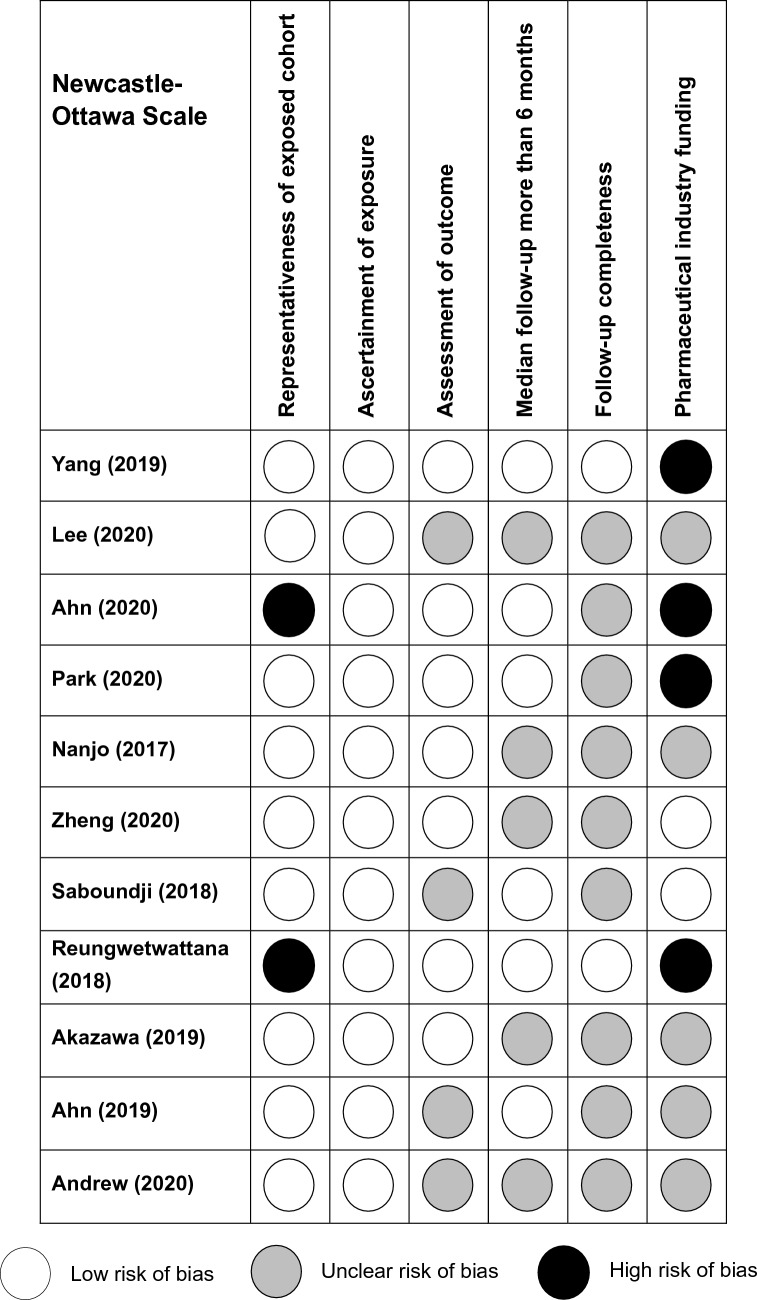
Modified Newcastle–Ottawa Scale of the included studies

The pooled one-year OS was 59% (95% CI 53% to 65%; I^2^ = 0%) in 233 patients from five studies (Fig. 3) [8, 13, 14, 17, 19]. Median PFS and OS were not aggregated and are presented narratively (Table 2). The median PFS was reported in seven studies [7, 8, 14, 16–18, 20], ranging from 3.7 to 17.3 months. The median OS was available in 5 studies [7, 8, 13, 14, 17], ranging from 11.0 to 18.8 months. In one study, osimertinib-treated patients were associated with improved OS compared with osimertinib-untreated patients (17.0 *versus* 5.0 months, HR = 0.36, 95% CI 0.28 to 0.47) [13]. In another study, the median iPFS was significantly longer in T790M-positive than T790M-negative patients who received osimertinib (15.6 *vs.* 7.0 months, *P* = 0.04) [16].

**Table 2:** Summary of extracted outcome data and pooled analysis

Study	LM ORR (%)			LM DCR (%)			PFS (moths)			OS (moths)		1-y OS rate (%)
	Median	95% CI		Median	95% CI		Median	95% CI		Median	95% CI	
Yang 2019	62 (23/37)	45–78		95 (35/37)	82–99		8.6	5.4–13.7		11.0	8.0–18.0	48.8
Lee 2020	NA	NA		NA	NA		NA	NA		17.0	15.1–18.9	61.8
Ahn 2020	55 (12/22)	32–76		91 (20/22)	NA		11.1	4.6-NR		18.8	6.3-NR	63.6
Park 2020	12.5 (5/40)	NA		92.5 (37/40)	NA		8.0	7.2-NR		13.3	9.1-NR	55.0
Nanjo 2017	NA)	NA		NA	NA		NA	NA		NR	NA	NA
Zheng 2020	38.2 (13/34)	NA		NA	NA		15.6^a^	4.0–17.2^a^		NA	NA	NA
							7.0^b^	4.0–10.0^b^				
Saboundji 2018	NA	NA		NA	NA		17.3	2.7–20.8		18.0	4.4–23.8	65.0
Reungwetwattana 2018	80 (4/5)	NA		100 (5/5)	NA		NA	NA		NA	NA	NA
Akazawa 2019	33.3 (2/6)	NA		83.3 (5/6)	NA		3.7	NA		NR	NA	NA
Ahn 2019	25 (10/40)	NA		90 (36/40)	NA		NA	NA		13.2	NA	NA
Andrew 2020	NA	NA		NA	NA		5.8	1.7–9.0		NA	NA	NA
Meta-analysis	42	24–59		90	85–94		NA	NA		NA	NA	59 (53–65)

CI: confidence interval; DCR: disease control rate; LM: leptomeningeal metastases; NA: not available; ORR: overall response rate; OS: overall survival; PFS: progression free survival

^a^T790M + patients

^b^T790M- patients

### Adverse events

Of the 11 included studies, AEs were reported in 4 prospective studies including 116 patients [7, 8, 14, 15]. The highest incidence of AEs of all grades was rash (53%, 95% CI 25% to 81%), followed by diarrhea (45%, 95% CI 29% to 61%), paronychia (35%, 95% CI 25% to 44%), decreased appetite (35%, 95% CI 13% to 57%), and dry skin (27%, 95% CI 18% to 35%). The pooled data of pneumonia with any grade and grade ≥ 3 were 5% (95% CI 0 to 9%) and 4% (95% CI 0 to 7%), respectively. AEs that led to osimertinib discontinuation were reported in 9 of 41 cases (22%) in one study and 1 of 22 cases (5%) in another study. There were no osimertinib-related deaths reported in the 4 studies. The details of common toxicities are presented in Table 3.

**Table 3:** Meta-analysis of common adverse events

Adverse events	No. of studies	Any grade				≥ Grade 3		
		Patients	Rates % (95% CI)	Heterogenicity (I^2^) (%)		Patients	Rates % (95% CI)	Heterogenicity (I^2^) (%)
Rash	3	45/94	48 (25–81)	88		1/94	1 (0–3)	0
Diarrhea	3	49/103	48 (29–61)	66		3/103	3 (0–5)	11
Paronychia	3	33/94	35 (25–44)	0		0/94	0 (0–2)	0
Decrease Appetite	3	38/103	37 (13–57)	84		3/103	3 (0–5)	17
Dry skin	3	28/103	27 (18–35)	0		0/103	0 (0–2)	0
Pneumonia	3	6/94	6 (0–8)	6		4/94	4 (0–7)	0
Nausea	2	22/81	27 (8–45)	75		1/81	1 (0–4)	0
Stomatitis	2	11/81	14 (6–21)	0		1/81	1 (0–4)	0

### Study quality assessment

The quality of the included studies was evaluated by modified NOS (Fig. 3). Four studies reported pharmaceutical industry funding [6–8, 14]. The FLAURA [6] and AURA studies [7] were considered to have low representativeness (high risk) because both studies only enrolled LM patients with stable neurological conditions. All studies ascertained the usage of osimertinib. Median follow-up was more than 6 months in 6 studies [6–8, 14, 17, 19]. Only 2 studies [6, 8] reported the percentage of patients lost to follow-up. Overall, 2 studies [8, 14] were at high risk of bias in 1 criterion, and 2 studies were at high risk of bias in 2 criteria [6, 7].

## Discussion

In this pooled analysis, we included 11 studies consisting of 353 patients, and the pooled results showed that the ORR, DCR, and one-year OS rate of osimertinib for the treatment of EGFR-mutant NSCLC with LM were 42% (95% CI 24% to 59%), 93% (95% CI 88% to 97%), and 59% (95% CI 53% to 65%), respectively. The ORR data were numerically lower than another pooled study of osimertinib for advanced NSCLC with metastasis to any site (ORR 79%, 95% CI 75–84%) [21], and lower than two meta-analyses of osimertinib for NSCLC with CNS metastases (mainly brain metastases, ORR was 64% and 70%, respectively) [11, 22]. Nonetheless, considering the refractory characteristics of LM, an ORR of 42% was acceptable or even satisfactory when compared with cytotoxic agents, radiotherapy or first- and second-generation EGFR TKIs [23–25].

The incidence of LM is considerably higher in EGFR-mutated NSCLC patients than EGFR wild-type ones (9.4% *vs.* 1.7%, *P* < 0.001) [26]. The third-generation TKI osimertinib was a favorable treatment option for patients with LM after resistance to first- or second-generation EGFR TKIs for two reasons. First of all, osimertinib is effective against the EGFR T790M mutation, which accounts for approximately 50% of the resistance from first- or second-generation EGFR TKIs [27]. Secondly, osimertinib is associated with a superior blood–brain barrier penetration capacity than other EGFR TKIs [6, 28]. Notably, disease progression in many patients was confined to LM with stable extracranial disease attributing to the poor penetration ability of gefitinib or erlotinib to the CNS system; whereas osimertinib is particularly highlighted for these patients. For treatment-naïve NSCLC LM patients, osimertinib is also recommended as an effective first-line treatment. In the FLAURA study, five patients in the osimertinib arm with LM, four of whom had a complete radiographic response and one had radiographic non-CR, non-PD response [6].

Whether increased doses (*e.g.*, 160 mg) could enhance the efficacy of osimertinib for LM is indeed a question deserves further discussions. In a phase II study including forty NSCLC patients with LM treated with 160 mg osimertinib, a 92.5% disease control rate and 12.5% complete response rate according to RECIST 1.1, a median PFS of 8.0 months, and median OS of 13.3 months were reported [14]. These data were encouraging and repeated by the BLOOM phase I study (ORR 62%, DCR 78%, PFS 8.6 months, and OS 11.0 months). The clinical use of osimertinib 80 mg once daily in patients with LM is effective and widely recommended. A retrospective study from Zheng et al*.* reported an LM ORR of 38.2% (13/34), and the median intracranial PFS was 15.6 months for EGFR T790M mutation patients who received 80 mg Osimertinib [16]. The AURA phase I study compared a wide range of osimertinib doses (20–240 mg) but did not reveal significant difference in efficacy, *e.g.*, 80 mg *vs.* 160 mg [29]. In our subgroup analysis, the pooled ORR was 47% for patients treated with 80 mg osimertinib, compared with 37% for patients treated with 160 mg osimertinib. To be noticed, studies of osimertinib 80 mg were either retrospective cohorts or studies with a small number of subjects (n = 13), or retrospective analyses of FLAURA and AURA series, which included only LM patients with stable neurological conditions. Further prospective studies for dosage specified efficacy and safety profiles of osimertinib are highly warranted.

The tolerance and safety are critical issues for NSCLC patients with LM since many patients have neurological impairment symptoms and progressively worsening. In our pooled analysis, rash (53%) and diarrhea (45%) of any grades were the most frequently reported AEs induced by osimertinib. This is in accordance with another meta-analysis on osimertinib for the treatment of advanced NSCLC with metastasis to any site (42% rash, 44% diarrhea). In the present study, pneumonia (5%) was the most frequently reported grade 3 or higher AE. No new safety concerns were identified in this study. No osimertinib-related death event was reported in the 4 included studies. The BLOOM study reported that all patients had at least 1 AE, 27 patients (66%) had an AE grade of 3 or higher, and 10 (24%) had AEs possibly causally related to osimertinib, as judged by the investigator. EGFR TKI-induced intestinal lung disease and pneumonia were frequent fatal AEs in a study based on 53 cohorts of 9569 participants [30].

Several limitations must be appreciated in this study. Among the first was the relatively small patient size in most included studies. Ten out of eleven included studies enrolled less than 50 patients. Second, all included studies were retrospective (n = 6) or phase I/II prospective (n = 5) studies, and no phase III randomized control trials were currently available. Third, the criteria of LM response assessment differed with different reports. Among the 11 studies, 3 studies defined treatment response by RANO-LM, 4 studies by RECIST or radiological response, and 4 studies did not report definitions of LM response. There is still a lack of suitable response assessment criteria for LM. RECIST is widely used for solid tumors; however, it is not appropriate for LM assessment since the presence of LM in imaging is usually linear but not bulk. RANO-LM was proposed to specifically evaluate the response assessment of LM in 2016 by incorporating several factors: standardized neurological examination, cerebral spinal fluid (CSF) cytology or flow cytometry, and radiographic evaluation [31]. RANO-LM is superior to RECIST for the response assessment of LM however still needs to be optimized [32]. Fourth, a meta-analysis of median PFS and OS was not conducted due to insufficient data available. Although we have previously shown that EGFR-mutated LM patients are likely to gain limited benefits from WBRT [2], the potential role of osimertinib in combinations with other treatment modalities such as WBRT or intrathecal chemotherapy for specific EGFR-mutated patients was yet to be elucidated, *i.e.*, co-existed with brain metastasis [33] or recurrent LM patients.

In conclusion, our systematic review and meta-analysis found that osimertinib showed meaningful CNS efficacy in terms of radiologic response and a manageable safety profile in patients with LM from EGFR-mutant NSCLC.

### Supplementary Information

**Additional file 1:** **Figure S1.** Funnel plot of potential publication bias of ORR, DCR and one-year survival.

## Data Availability

Not applicable.

## Abbreviations

AEs: Adverse events
BBB: Blood–brain barrier
CI: Confidence interval
CNS: Central nervous system
CSF: Cerebrospinal fluid
EGFR: Epidermal growth factor receptor
TKIs: Tyrosine kinase inhibitors
LM: Leptomeningeal metastases
NOS: Newcastle–Ottawa Scale
ORR: Objective response rate
OS: Overall survival
RECIST: Response Evaluation Criteria in Solid Tumors
NSCLC: Non-small cell lung cancer
PFS: Progression-free survival
PRISMA: Preferred Reporting Items for Systematic Reviews and Meta-analysis
RANO: Response Assessment in Neuro-Oncology
