# Supplementary figures and images for: Efficacy and safety of osimertinib for leptomeningeal metastases from EGFR-mutant non-small cell lung cancer: a pooled analysis
Source: Eur J Med Res. 2023 Aug 4;28:267. doi: 10.1186/s40001-023-01219-y (PMC10403821; doi:10.1186/s40001-023-01219-y)

# ORR

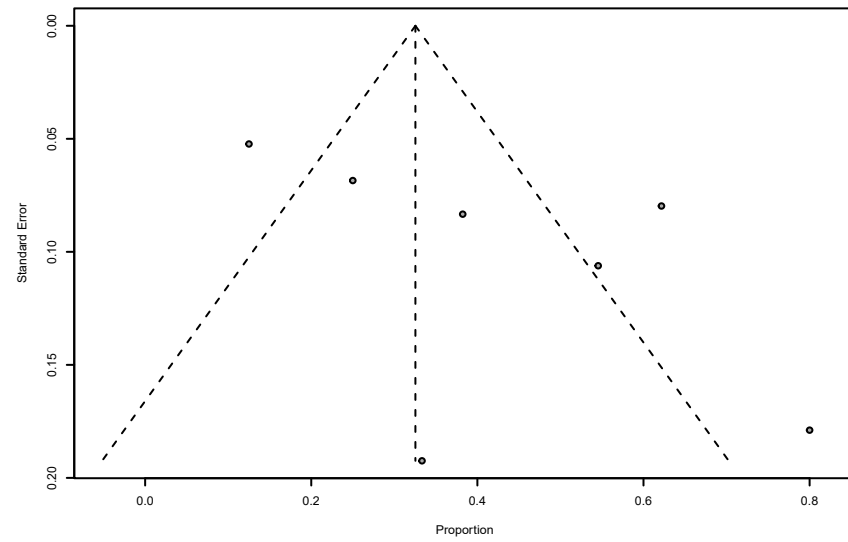

# DCR

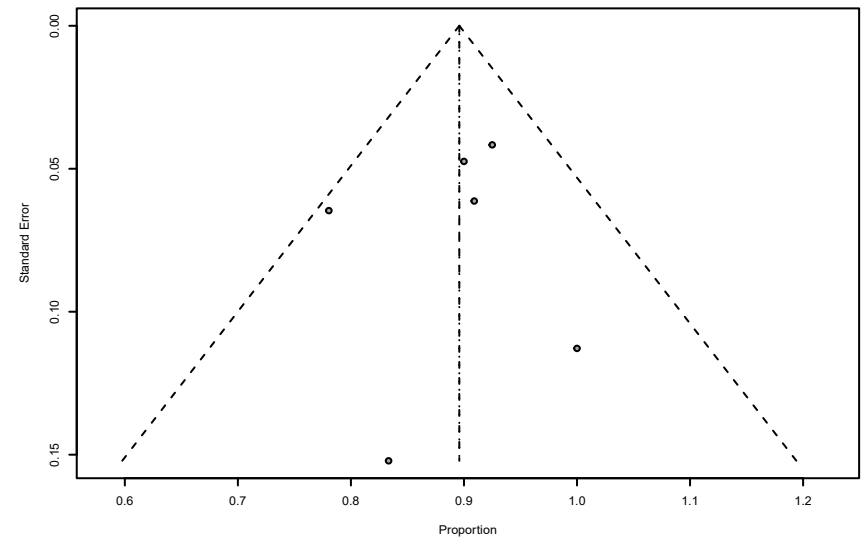

# 1-y OS

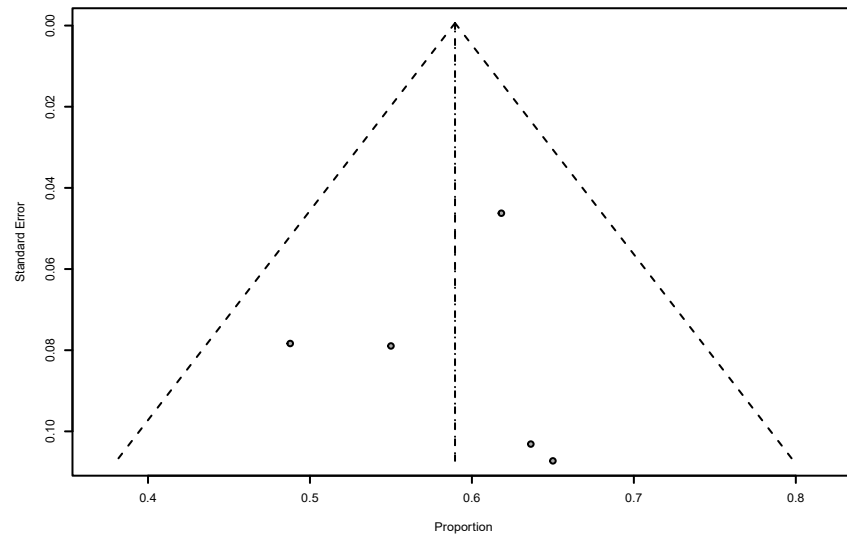

Supplement: Supplementary file 1 — Additional file 1: Figure S1. Funnel plot of potential publication bias of ORR, DCR and one-year survival. [file 40001_2023_1219_MOESM1_ESM.pdf]
